# Cerebral Hemodynamic Correlates of Transcutaneous Auricular Vagal Nerve Stimulation in Consciousness Restoration: An Open-Label Pilot Study

**DOI:** 10.3389/fneur.2021.684791

**Published:** 2021-07-15

**Authors:** Yutian Yu, Yi Yang, Shuoqiu Gan, Shengnan Guo, Jiliang Fang, Shouyan Wang, Chunzhi Tang, Lijun Bai, Jianghong He, Peijing Rong

**Affiliations:** ^1^Acupuncture Department, Beijing Shijitan Hospital, Capital Medical University, Beijing, China; ^2^Ninth School of Clinical Medicine, Peking University, Beijing, China; ^3^Institute of Acupuncture and Moxibustion, China Academy of Chinese Medical Sciences, Beijing, China; ^4^Department of Neurosurgery, Beijing Tiantan Hospital, Capital Medical University, Beijing, China; ^5^Department of Neurosurgery, PLA Army General Hospital, Beijing, China; ^6^The Key Laboratory of Biomedical Information Engineering, Ministry of Education, Department of Biomedical Engineering, School of Life Science and Technology, Xi'an Jiaotong University, Xi'an, China; ^7^Department of Medical Imaging, The First Affiliated Hospital of Xi'an Jiaotong University, Xi'an, China; ^8^Department of Radiology, Guang'anmen Hospital, China Academy of Chinese Medical Sciences, Beijing, China; ^9^Institute of Science and Technology for Brain-Inspired Intelligence, Fudan University, Shanghai, China; ^10^Clinical Medical College of Acupuncture, Moxibustion and Rehabilitation, Guangzhou University of Chinese Medicine, Guangzhou, China

**Keywords:** functional magnetic resonance imaging (fMRI), arterial spin labeling (ASL), cerebral blood flow (CBF), transcutaneous auricular vagal nerve stimulation (taVNS), disorders of consciousness (DOC), responded to auditory stimuli (RtAS), non-responded to auditory stimuli (nRtAS)

## Abstract

This study aimed to preliminarily illustrate the cerebral hemodynamic correlates of transcutaneous auricular vagal nerve stimulation (taVNS) in consciousness restoration. Arterial spin labeling (ASL) was adopted with functional magnetic resonance imaging (fMRI) to measure cerebral blood flow (CBF) changes before and after taVNS in 10 qualified patients with disorders of consciousness (DOC). Before taVNS, five patients responded to auditory stimuli (RtAS), and five did not respond to auditory stimuli (nRtAS). The RtAS DOC patients obtained favorable prognoses after the 4-week taVNS treatment, whereas the nRtAS ones did not. Simultaneously, taVNS increased CBF of multiple brain regions in the RtAS DOC patients, but hardly in the nRtAS ones. In conclusion, the preserved auditory function might be the prior key factor of the taVNS responders in DOC patients, and taVNS might alleviate RtAS DOC by activating the salience network, the limbic system, and the interoceptive system.

## Introduction

Emergency healthcare and reanimation techniques have decreased the mortality of patients with severe traumatic brain injury (TBI) and hypoxic-ischemic encephalopathy (HIE) significantly in recent decades. However, some patients with TBI or HIE manifest with very poor prognoses and finally suffer from disorders of consciousness (DOC), a medical condition changed from complete self-awareness to inhibited or absent self-awareness and arousal (1), either temporary or permanent. Increasing numbers of DOC patients are laying an enormous burden on families, economies, and societies worldwide. Thus, the exploration of a novel method to restore consciousness is urgently needed.

Although new neuromodulation techniques, such as transcranial direct current stimulation (tDCS) (2), transcranial magnetic stimulation (TMS) (3), and low intensity focused ultrasound pulse (LIFUP) (4), have been introduced (5), deep brain stimulation (DBS), and spinal cord stimulation (SCS) remain two mostly employed neurostimulation techniques for DOC patients and show some promises in restoring consciousness (6, 7). However, high costs, complicated processes, and potential surgical side effects limited their applications.

Corazzol et al. applied vagal nerve stimulation (VNS) to treat a patient lying in the vegetative state (VS) for 15 years following TBI (8). After 1 month of stimulation, clinical examination revealed reproducible, stable improvements in general arousal, sustained attention, body motility, and visual pursuit. Scores on the Coma Recovery Scale-Revised (CRS-R) test were improved, indicating a transition from a vegetative to minimally conscious. The results challenged the belief that DOC persisting longer than 12 months is irreversible (9). However, the same as DBS and SCS, VNS is expensive and invasive with irreversible implants. The disadvantages block its clinical application in more DOC patients.

The vagal nerve carries somatic and visceral efferents and afferents distributed throughout the brain, either monosynaptically or *via* the nucleus tractus solitarii (NTS) (10). The vagal nerve has a branch of afferent projections in the auricular concha and external ear channels (11). Thus, transcutaneous auricular vagal nerve stimulation (taVNS) was developed based on this anatomical characteristic (12, 13) and was previously found to produce comparable efficacy with classic VNS (14), which means taVNS is a promising form of classic VNS (15).

Before the French team's publication (8), our team also has a peer-reviewed case report on the beneficial effects of taVNS on the consciousness level of a 73-year-old female single patient who developed into DOC after cardiopulmonary resuscitation for 50 days (16). After a 4-week taVNS treatment, her CRS-R scores rose from 6 to 13, and her diagnosis was changed from VS to minimally conscious state (MCS). The BOLD functional magnetic resonance imaging (fMRI) outcomes also showed improved brain functional connectivity (FC). This was the first case of taVNS in a DOC patient and the first report of encouraging results from clinical conditions to brain FC.

After the case report, we then showed that only auditory-function-preserved patients with DOC are reversible by taVNS (17). In the same survey, we also focused on arterial spin labeling (ASL) with fMRI in the brain of these DOC patients, which uses magnetically labeled arterial blood water protons as an endogenous tracer and is a non-ionizing and non-invasive measurement of cerebral blood flow (CBF) (18, 19), and the most effective approach uses magnetically labeled arterial blood water (20). Arterial spin labeling perfusion is commensurate with other more invasive methods such as PET and dynamic susceptibility contrast-enhanced MRI (DSC-MRI) perfusion (21), with higher accuracy and acceptance, and without specific ethics requirements in humans (22, 23). For DOC patients, especially the ones in VS, the increment of CBF is the basis of their brain functional recovery (24). Nevertheless, the ASL-fMRI results of the study were neither sufficiently illustrated nor adequately discussed previously. Thus, in this study, we aimed to preliminarily illustrate the cerebral hemodynamic correlates of taVNS in consciousness restoration.

## Methods

### Ethics

This is an open-label pilot study within a clinical trial (Trial registration: ChiCTR-INR-16008745). The study was reviewed and approved by the Ethics Committee of the Institute of Acupuncture and Moxibustion, China Academy of Chinese Medical Sciences (Approval Number: 2016062001). Written informed consent to participate in this study and to publish the work was provided by the legal guardians of the patients.

### Participants and Scales Assessing

Patients were recruited from the Department of Neurosurgery, PLA Army General Hospital, Beijing, China, for this study. Inclusion criteria were patients in VS, MCS, and coma following severe brain damage after the acute brain insult for at least 2 days. Patients were excluded when there was a contraindication for MRI (e.g., presence of ferromagnetic aneurysm clips and pacemakers), MRI acquisition under sedation or anesthesia, uncertain clinical diagnosis (25), and data could not be further processed in the further ASL-fMRI analysis.

The patients' consciousness states were assessed twice, before (T0) and after (T1) the taVNS treatment, using the JFK Coma Recovery Scale (JFK CRS-R) (26), which includes six subscales addressing auditory, visual, motor, oromotor, communication (language), and arousal processes. The elapsed time between eligibility and baseline assessments was within 24 h.

Also, their prognoses were judged *via* the Glasgow Outcome Scale (GOS). Glasgow Outcome Scale provides a measurement of outcome ranging from 1 to 5 (1, dead; 2, vegetative state/severe disability; 3, able to follow commands/unable to live independently/moderate disability; 4, able to live independently/unable to return to work or school; 5, good recovery/able to return to work or school) (7, 27). In this study, any GOS score ≤ 2 was defined as “unfavorable prognosis,” whereas a score from 3 to 5 was defined as “favorable prognosis” (7, 28, 29).

No other treatments, including drugs that could modify cortical excitability, were administered. And we followed the CONSORT checklist in this study.

### The taVNS Treatment

The points for taVNS were placed in the concha area, where there are principal vagal nerve branch distributions (11, 14, 30). Both the cymba concha (100% auricle branch of the vagal nerve) and the cavity of concha (45% auricle branch of the vagal nerve and 55% great auricular nerve) (11) were stimulated (Supplementary Figure 1). After the stimulation areas were sterilized, ear clips with plate electrodes were attached to the area (auricular concha) at the stimulation site (Supplementary Figure 1) of both ears. Stimulation parameters were adjusted according to our previous study (16): (1) density wave to 20 Hz, and pulse width to 0.5 ms; (2) intensity: 4–6 mA (this intensity will cause slight pain to the ears in conscious people, which is mostly tolerable). The treatment lasted 30 min continuously and was carried out twice a day (8:00 and 16:00) for 4 weeks.

### Functional MRI Data Acquisition

Patients received fMRI scanning sessions before and after the treatment. The elapsed time between the MRI scans and treatment was within 24 h. Most of the scans were carried out at around 15:00. A 3.0 T MR scanner (HD750, General Electronic Co., USA) was used for this study equipped with an eight-channel head coil. A wedge-shaped foam padding was used to minimize head motions.

Structure imaging included 3D-T1-weighted. Arterial spin labeling sequences were obtained during the eyes-open status. Patients' ASL sequences with significant motion degradation were excluded from the analysis.

The raw ASL images were acquired twice by three-dimensional ASL sequences (31–33). The 3D ASL, including M0 image and perfusion different image, was obtained with the parameters as follows: TR = 4,632 ms, TE = 10 ms, slice thickness = 4 mm, field of view (FOV) = 24 ×24 mm^2^, post labeling delay (PLD) = 1,500 ms. In addition, T1-weighted three-dimensional high-resolution structural images were obtained using a sagittal BRAin Volume imaging (BRAVO) sequence with TR = 7.8 ms, TE = 3.0 ms, TI = 600 ms, flip angle of 9°, and 186 slices with a voxel size of 1 ×1 ×1 mm^3^.

### ASL Pre-processing

Arterial spin labeling pre-processing used ASLtbx based on SPM12 (Statistical Parametric Mapping, available at www.fil.ion.ucl.ac.uk/spm/software/Spm12) on the Matlab platform (R2013b; Math Works, Natick, MA).

Before calculating the CBF map, the orientation of each 3D ASL and 3D T1 image should be reset to the center of the image matrix at the midpoint of the AC-PC line. The CBF (ml/100 g/min) map of 3D ASL was calculated using the mean Perf difference image, and M0wm (M0wm was extracted from a white matter mask) by using batch scripts provided in ASLtbx (34). Perfusion difference image was registered to individual T1 image so that they could be later normalized to Montreal Neurological Institute (MNI) template space for next smoothing. We normalized CBF within the ASL images to avoid major variations in CBF due to cardiac blood flow and pressure liabilities. The normalized CBF map was smoothed with an isotropic Gaussian filter with a full-width-at-half-maximum (FWHM) = 6 mm^3^ to filter noise for later group analysis. We then performed whole-brain voxel-wise analyses of the images within the general linear model framework using SPM12. The analyses were constrained to gray matter tissue only by thresholding the analysis mask to 40% of the mean gray matter image of our sample.

### ASL Statistics Analysis

After pre-processing, we extracted mean CBF from 92 regions of interest using the AAL parcellation toolbox. The following data analysis processing was conducted on SnPM13, alongside SPM12b installation. SnPM refers to an implementation of Statistical non-Parametric Mapping by Andrew Holmes and Tom Nichols (35). The voxel-wise comparison was used in SnPM to examine CBF increment from pre-taVNS to post-taVNS, with 5,000 permutations. Regions with significant CBF changes were defined as ROIs, and the ROI-based CBF change ratio [CBF change ratio = (post_CBF – pre_CBF)/pre_CBF] was calculated.

### Clinical Data Analysis

To compare the consciousness recovery state before and after the taVNS treatment, the patients' CRS-R total scores and six subscales' scores of each group were analyzed with *t*-tests, respectively. GraphPad Prism 6 software was used to analyze the data. Differences with *P* < 0.05 were considered statistically significant.

## Results

### Demographic Information

The demographic information was shown in Table 1. Briefly, we recruited seven males and three females, aged from 19 to 73 years old; three of them were with MCS, while seven of them were with VS. In these patients, five of them were caused by HIE, two by brain injuries due to traffic accidents, two by a cerebral hemorrhage, and one by brainstem hemorrhage.

**Table 1:** Patients' demographics and clinical characteristics.

**ID**	**State**	**Grouping before taVNS treatment**	**Gender**	**Age**	**Course**	**Cause**	**Prognosis**	**GOS**	**CRS-R (T0) before the taVNS treatment**	**CRS-R (T1) after the taVNS treatment**
P01	VS	RtAS	Male	41	10 days+	HIE	Favorable	3	A1V0M2O1C0Ar2	A2V3M2O1C0Ar2
P02	VS	nRtAS	Male	43	50 days+	HIE	Unfavorable	2	A0V0M2O1C0Ar2	A1V1M2O1C0Ar2
P03	VS	nRtAS	Male	31	90 days+	cerebral hemorrhage	Unfavorable	2	A0V0M2O1C0Ar2	A1V1M2O1C0Ar2
P04	MCS	RtAS	Male	23	90 days	HIE	Favorable	4	A2V3M2O1C0Ar2	A4V5M5O2C2Ar3
P05	MCS	RtAS	Female	27	300 days	Brain injuries due to traffic accident	Favorable	3	A3V3M3O1C0Ar3	A3V3M3O1C0Ar3
P06	MCS	RtAS	Male	42	50 days+	Cerebral hemorrhage	Favorable	5	A1V0M3O1C0Ar2	A4V1M6O3C2Ar3
P07	VS	nRtAS	Male	39	30 days+	Brainstem hemorrhage	Unfavorable	2	A0V0M2O0C0Ar0	A0V0M2O0C0Ar0
P08	VS	nRtAS	Male	29	60 days+	HIE	Unfavorable	2	A0V0M1O0C0Ar2	A1V2M2O0C0Ar2
P09	VS	nRtAS	Female	19	15 days	Brain injuries due to traffic accident	Unfavorable	2	A0V1M2O0C0Ar1	A1V1M2O1C0Ar2
P10	VS	RtAS	Female	73	90 days+	HIE	Favorable	3	A1V1M1O1C0Ar2	A3V3M3O2C0Ar2

*VS, vegetative state; MCS, minimally conscious state; RtAS, response to auditory stimulus; nRtAS, non-response to auditory stimulus; HIE, hypoxic-ischemic encephalopathy; GOS, Glasgow Coma scale; CRS-R, Coma Recovery Scale-Revised. The subscales for the CRS-R are Auditory Function (A), Visual Function (V), Motor Function (M), Oromotor Function (O), Communication (C), and Arousal (Ar)*.

### Clinical Characteristics

According to the first auditory score (Table 1), five patients fell into the responded to auditory stimuli (RtAS) group [subscale Auditory Function (A) ≥ 1: Auditory startle], and the other five fell into the non-responded to auditory stimuli (nRtAS) group [subscale Auditory Function (A) = 0: None].

Through the GOS assessment (Table 1) (7, 29), after the taVNS treatment, five patients can be classified as favorable prognoses (GOS > 2), while the other five patients can be classified as unfavorable prognoses (GOS ≤ 2).

The data illustrated that patients, who responded to auditory stimuli [RtAS, CRS-R (T0) subscale Auditory Function (A) ≥ 1] before the taVNS treatment, were going to have favorable prognoses (GOS > 2) after the treatment. Simultaneously, patients, who did not respond to auditory stimuli [nRtAS, CRS-R (T0) subscale Auditory Function (A) = 0] before the taVNS treatment, were going to have unfavorable prognoses (GOS ≤ 2) after the treatment.

**Figure 1:**
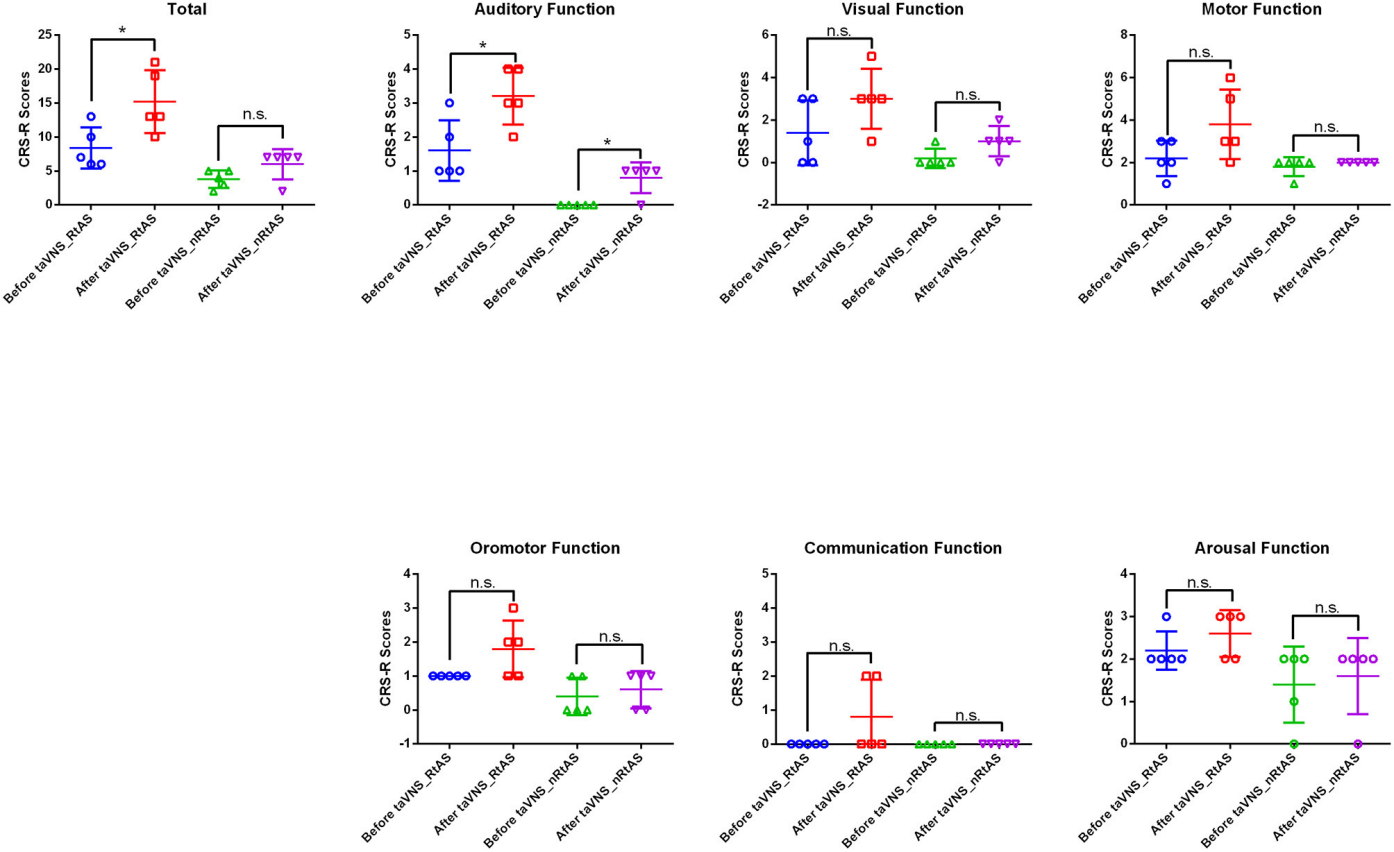
Patients' CRS-R total and subitems scores before and after taVNS. *P < 0.05, before taVNS vs. after taVNS; n.s., not significant; *P* > 0.05; before taVNS vs. after taVNS.

All patients' CRS-R subitems and total scores before and after the taVNS treatment were presented in Table 1 and Figure 1. The data showed that taVNS only improved the total scores of the RtAS group significantly (*P* < 0.05). As a result, we reported that only the RtAS DOC patients were responsive to taVNS (17).

### CBF Changes

**Figure 2:**
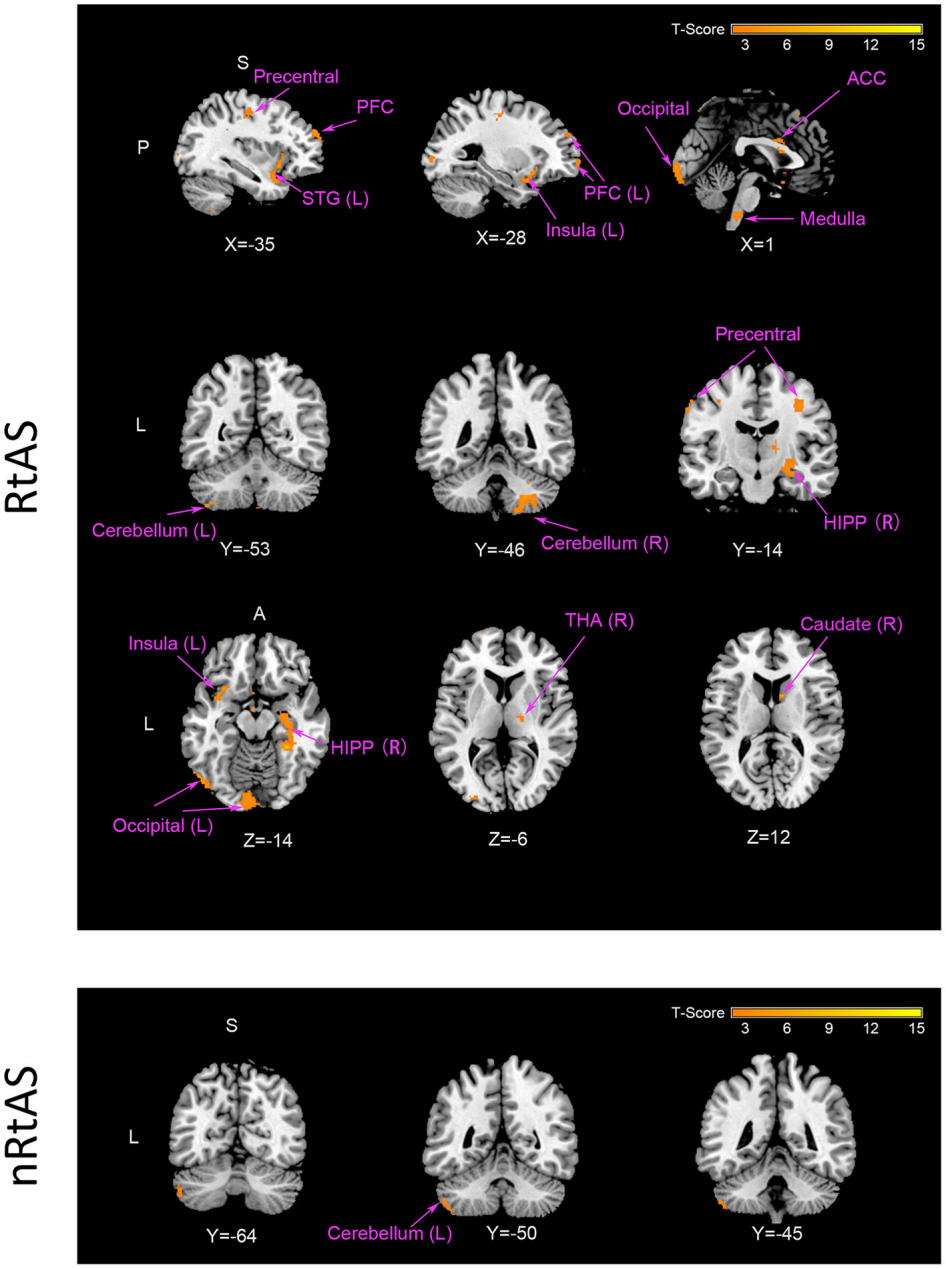
CBF increment by taVNS. The RtAS group: taVNS increased the RtAS DOC patients' CBF in this study in many brain regions presented in this figure: STG (L) of temporal lobe; PFC (L) including SFG (L), MFG (L), and IFG (L); medulla; cerebellum (L) and cerebellum (R); precentral; caudate (R); HIPP (R); insula (L); occipital (L); THA (R). Images displayed in Montreal Neurological Institute standard stereotaxic space, and coordinates are provided for each slice. STG (L), left superior temporal gyrus; PFC (L), left prefrontal cortex including left superior frontal gyrus [SFG (L)], left middle frontal gyrus [MFG (L)], and left inferior frontal gyrus [IFG (R)]; cerebellum (L), left cerebellum; cerebellum (R), right cerebellum; precentral, precentral gyrus; caudate (R), right caudate; HIPP (R), right hippocampus; insula (L), left insula; occipital (L), left occipital lobe; THA (R), right thalamus. The nRtAS group: taVNS increased the nRtAS DOC patients' CBF only in the left cerebellum. Images displayed in Montreal Neurological Institute standard stereotaxic space and coordinates are provided for each slice. Cerebellum (L), left cerebellum. Uncorrected *P* < 0.05, *T* > 1.97.

As shown in Table 2 and Figure 2, taVNS increased CBF of multiple brain regions in the RtAS DOC patients; the CBF increment in the nRtAS group with the taVNS treatment is relatively weak, which was merely prominent in the left cerebellum. Cerebral blood flow changes ratio graphs, and some ROI-based CBF changes between pre-taVNS and post-taVNS in the RtAS group can be seen in Supplementary Figure 2 and Supplementary Table 1.

**Table 2:** CBF increased brain regions by taVNS.

	**CBF improved regions**	**MNI (mm)**			**T-score**	**Voxel size**
		**X**	**Y**	**Z**		
RtAS	Thalamus (R)	18.29	−11.97	3.22	2.90	76
	Superior Temporal Gyrus (L)	−35.62	6.64	−25.75	5.10	81
	Superior Frontal Gyrus (L)	−24.93	66.99	−14.26	5.03	262
	Precentral Gyrus (R)	42.35	−5.83	45.45	3.14	53
	Precentral Gyrus (L)	−64.67	−4.73	42.94	3.86	178
	Postcentral Gyrus (L)	−32.50	−16.64	46.28	2.54	54
	Hippocampus (R)	33.16	−43.34	−11.74	10.16	64
	Orbital Gyrus (L)	−8.07	31.81	−41.40	4.57	131
	Middle Temporal Gyrus (R)	73.64	−1.92	−16.82	3.06	92
	Middle Frontal Gyrus (R)	29.52	−1.48	47.09	2.43	71
	Middle Frontal Gyrus (L)	−50.28	48.99	7.57	3.49	227
	Midbrain (R)	15.89	−19.31	−18.89	5.49	188
	Medulla (R)	2.78	−39.72	−43.98	10.61	64
	Medulla (L)	0.62	−40.00	−45.80	2.79	116
	Occipital Lobe (R)	5.66	−100.33	1.17	3.38	77
	Occipital Lobe (L)	−2.87	−95.73	4.40	3.88	113
	Insula (L)	−31.20	19.01	−12.56	3.93	112
	Inferior Frontal Gyrus (L)	−31.32	8.96	−24.28	6.67	92
	Cerebellum (R)	15.61	−48.40	−46.67	22.86	811
	Cerebellum (L)	−39.97	−61.17	−45.69	9.80	147
	Caudate (R)	11.86	9.38	6.32	5.66	115
	Caudate (L)	−5.26	15.76	7.70	3.97	83
	Lentiform Nucleus (R)	33.11	−15.89	−10.23	4.06	107
	Lentiform Nucleus (L)	−33.14	−19.69	−4.75	2.58	69
nRtAS	Cerebellum (L)	−54.70	−71.41	−28.72	3.79	61

*R, right; L, left; MNI, Montreal Neurological Institute. Voxel <50 were excluded in this study*.

## Discussion

VNS (8) and taVNS (16) have been identified as therapeutic strategies for DOC (5). Signs of consciousness recovery after taVNS were recorded in some severe TBI patients (36). It was also suggested that taVNS is a feasible, safe, and effective tool for DOC patients (37).

Initially, we designed a clinical trial, planning to use taVNS and transcutaneous non-vagal nerve stimulation (tnVNS, as a sham control group) in DOC patients. However, previous studies (36, 37) and this one found that only a portion of DOC patients is responsive to taVNS, which implied that designing a tnVNS group is not yet necessary presently (and that the clinical trial might have failed); however, finding the prior key factor(s) of the taVNS responders in DOC patients is more valuable and instructive to future studies. It has been reported that the auditory network FC has good correspondence with the level of consciousness (25), which is also estimated to be capable of predicting the prognoses of DOC (38). Intriguingly, we found that only RtAS DOC patients were responsive to taVNS (17). Therefore, the evidence provides a possible answer that the preserved auditory function might be the key factor.

The discovery of the key factor is indeed accidental. We originally assumed that all DOC patients are responsive to taVNS. Thus, this work formerly intended to reveal more detailed neuroimaging evidence of how taVNS alleviates DOC. And the results are also fortuitous and interesting.

Previous neurophysiologic taVNS/fMRI studies in healthy subjects majorly focused on the BOLD signal (15, 39–41). Because they applied different stimulating methods (sites and parameters), they have different findings. In this study, we deployed taVNS in DOC patients, in which both the cymba concha and the cavity of concha (11) were stimulated (Supplementary Figure 1). We found some overlapping brain regions in RtAS DOC patients with the previous studies, such as the thalamus, the caudate, the insula, and the frontal cortex. Nevertheless, this study majorly focused on the altered ASL signal in DOC patients. Meanwhile, neural networks and systems based on the brain regions need to be interpreted to deepen the study's insight. In the former two works (8, 16), the primary discoveries were the default mode network (DMN), the thalamo-cortical network, and the centro-posterior network. A hypothesis article has proposed three new possible neural networks (42): the external fronto-parietal network (ExN), the salience network, and the Mesocircuit model. This work provides some new evidence and may confirm the salience network hypothesis.

Impaired consciousness was proportional to the reduction in mean CBF regardless of pathology types (43). Therefore, increment in CBF is the basis of consciousness recovery. The results of this study showed that taVNS increased CBF of multiple brain regions in the RtAS DOC patients, but hardly in the nRtAS ones (Table 2 and Figure 2). Here, we disclose the details.

### The First Level: Through the Vagal Nerve, taVNS Might Modulate the Salience Network and the Limbic System

The afferent vagal fibers connect to the NTS in the medulla, which in turn projects connections to other locations in the brain (44, 45). Previous studies confirmed that taVNS precisely activated the NTS in healthy subjects (41). The results of this study showed that taVNS increased CBF of the medulla in the RtAS DOC patients, which indicated that taVNS might also modulate the NTS directly in these patients. With the modulation of NTS, the peripheral nerve stimulations can pass through the vagal nerves and reach the thalamus (46). The thalamus is the large mass of gray matter in the dorsal part of the brain with several functions such as relaying of sensory signals, including motor signals, to the cerebral cortex (47, 48) and the regulation of consciousness, sleep, and alertness, which also plays a vital role in arousal and awareness (49). Through the medulla and the thalamus, taVNS might modulate patients' left insula, which plays an essential role in consciousness (50). It has been proposed that primates possess a unique mapping of autonomic interoceptive information within the insula that forms the substrate of conscious feelings (51). The insula is also one of the core brain regions that anchor the salience network (52), which segregates the most relevant internal and extrapersonal stimuli (52) and is associated with individual differences in interoceptive accuracy (53). This study showed that taVNS increased CBF of the insula in the RtAS DOC patients, indicating that taVNS might activate these patients' consciousness by improving their salience network connectivity (42). Cerebral blood flow upregulations of the insula directly accentuated some limbic-related areas such as the hippocampus, which is also recognized as a critical structure for autonoetic consciousness (54–56). taVNS also increased CBF of other limbic-related regions such as the caudate, the middle temporal gyrus (MTG), the orbitofrontal cortex (OFC), the inferior frontal gyrus (IFG). Significantly, the IFG is involved in evaluating linguistic, interoceptive, and emotional information (57), including visuospatial attention (58), and the improvement of these cognitive functions by taVNS might be one basis of consciousness recovery in these RtAS DOC patients.

### The Second Level: Through the Up-Conducting Pathway of the Interoceptive System, taVNS Might Modulate the Cerebral Cortex

The improved thalamic fundamental metabolic level leads to the CBF upregulations of the cerebral cortex [somatosensory cortex (occipital lobe, superior temporal gyrus—STG, and MTG) and executive control cortex (prefrontal areas)] through the up-conducting pathway of the interoceptive system (51, 59). Meanwhile, the improved insula fundamental metabolic level also leads to metabolic upregulations of the somatosensory cortex and the prefrontal areas, which are also involved in the interoceptive system (51, 60). The interoceptive system is crucial for maintaining homeostatic conditions (61) in the body and, potentially, aiding in self-awareness (62) and is fundamental in human emotional well-being (59) and consciousness (51). Traceabily, interoceptive signals are transmitted to the brain *via* multiple pathways, and the vagal nerve is one of them (51). Thence, taVNS might trigger the consciousness restoration effects in the RtAS DOC patients by activating the interoceptive system. It is worth mentioning that the STG is not merely involved in auditory processing, including language, but also has been implicated as a critical structure in social cognition (63, 64). Therefore, CBF upregulations of the STG by taVNS might also help to restore consciousness by improving these RtAS DOC patients' cognitive functions.

### The Third Level: taVNS Might Modulate the Thalamo-Cortical Loop

Previously, a consensus has been reached that disconnections in long-range thalamo-cortical pathways are involved in DOC's situation (65). The caudate is one of the structures that make up the dorsal striatum, a basal ganglia component (66). The increment of CBF in these RtAS DOC patients' caudate illustrated that taVNS might modulate the (ganglia-)thalamo-cortical loop (67), which might reconnect the very disconnections.

## Limitations

Considering the small sample size and the lack of a control group, we should interpret our data cautiously. One of the limits of this open-label pilot study is that there were only 10 qualified DOC patients enrolled, with only 5 being responsive to taVNS, which can hardly illustrate all detailed mechanisms of the consciousness restoration by taVNS. Moreover, before taVNS, patients in the RtAS group already had better clinical conditions than those in the nRtAS group: three patients of the RtAS group were in MCS, and two were in VS, whereas all five of the nRtAS subjects were in VS (Table 1); this indicated that the observed differences in response between RtAS and nRtAS might have been significantly influenced by differences in these patients' baseline clinical conditions. It is also unclear whether a DOC patient being responsive to auditory stimuli would respond to any extended procedures, which presumably include auditory stimuli rather than the actual procedure, such as taVNS. Also, the benefits of taVNS require time to emerge in DOC patients; however, we set the duration of the taVNS treatment for 4 weeks in this study (17), limiting its effects for the patients (the French team monitored the effects of VNS for more than 6 months in that particular case) (5, 8). In addition, to map more detailed mechanisms, other monitoring methods, such as functional near-infrared spectroscopy (fNIRS), electroencephalography (EEG), and magnetoencephalography (MEG), need to be considered in upcoming studies. Eventually, physiological parameters, such as blood pressure, pulse rate, heart rate variability, and the baroreflex, need to be tested in the future to measure tolerability and parasympathetic activity.

## Conclusion

This study newly demonstrated that taVNS might primarily activate the salience network, the limbic system, and the interoceptive system, which better illustrates how taVNS alleviates RtAS DOC than the previous studies. The results also indicated that the preserved auditory function might be the prior key factor of the taVNS responders in DOC patients. Therefore, future time-limited controlled trials applying taVNS and tnVNS (as a sham control group) on DOC patients should probably avoid enrolling the nRtAS ones.

## Data Availability Statement

The original contributions presented in the study are included in the article/Supplementary Material, further inquiries can be directed to the corresponding author/s.

## Ethics Statement

The studies involving human participants were reviewed and approved by the Ethics Committee of the Institute of Acupuncture and Moxibustion, China Academy of Chinese Medical Sciences. Written informed consent to participate in this study was provided by the patients legal guardians.

## Author Contributions

YYu and YYa designed the study and drafted the manuscript. YYa and JH recruited participants and performed data collection. SGa and LB analyzed the data. PR, CT, JF, and SW provided resources and general assistance. YYu and SGu localized the electrodes. All authors provided feedback on the manuscript, contributed to the article, and approved the submitted version.

## Conflict of Interest

The authors declare that the research was conducted in the absence of any commercial or financial relationships that could be construed as a potential conflict of interest.
