# Supplementary material for: Cerebral Hemodynamic Correlates of Transcutaneous Auricular Vagal Nerve Stimulation in Consciousness Restoration: An Open-Label Pilot Study
Source: Front Neurol. 2021 Jul 15;12:684791. doi: 10.3389/fneur.2021.684791 (PMC8319239; doi:10.3389/fneur.2021.684791)
Supplement: Supplementary file 1 [file Data_Sheet_1.docx]

**Supplementary Material: Restoring consciousness with transcutaneous auricular vagal nerve stimulation monitored by ASL-fMRI**

Yutian Yu, Yi Yang, Shuoqiu Gan, Shengnan Guo, Jiliang Fang, Shouyan Wang, Chunzhi Tang, Lijun Bai, Jianghong He, Peijing Rong

**Correspondence**

Yutian Yu

E-mail: yutianyu@bjsjth.cn

Acupuncture Department, Beijing Shijitan Hospital, Capital Medical University, Beijing, China.

Ninth School of Clinical Medicine, Peking University, Beijing, China.

Yi Yang

E-mail: yangyi_81nk@163.com

Department of Neurosurgery, Beijing Tiantan Hospital, Capital Medical University, Beijing, China.


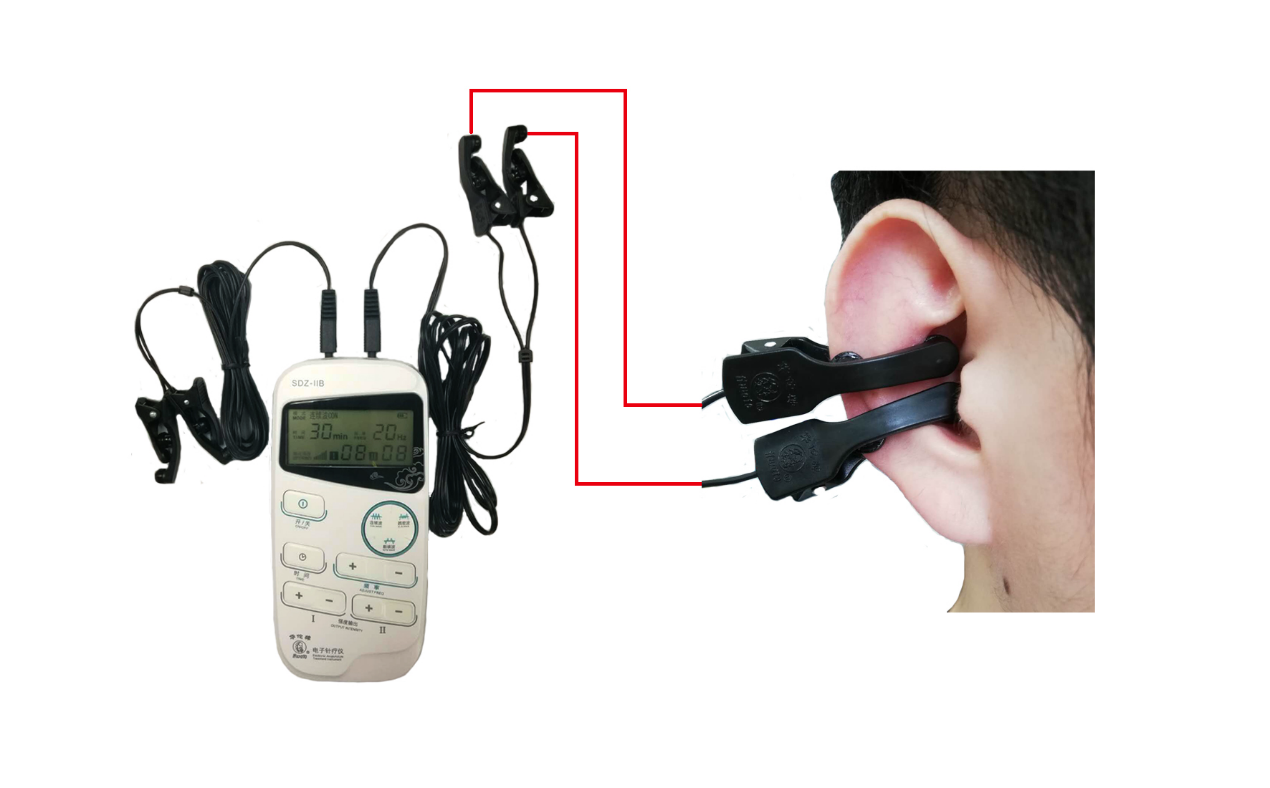


**Supplementary Figure 1 The stimulator and the ear clips placement**

**
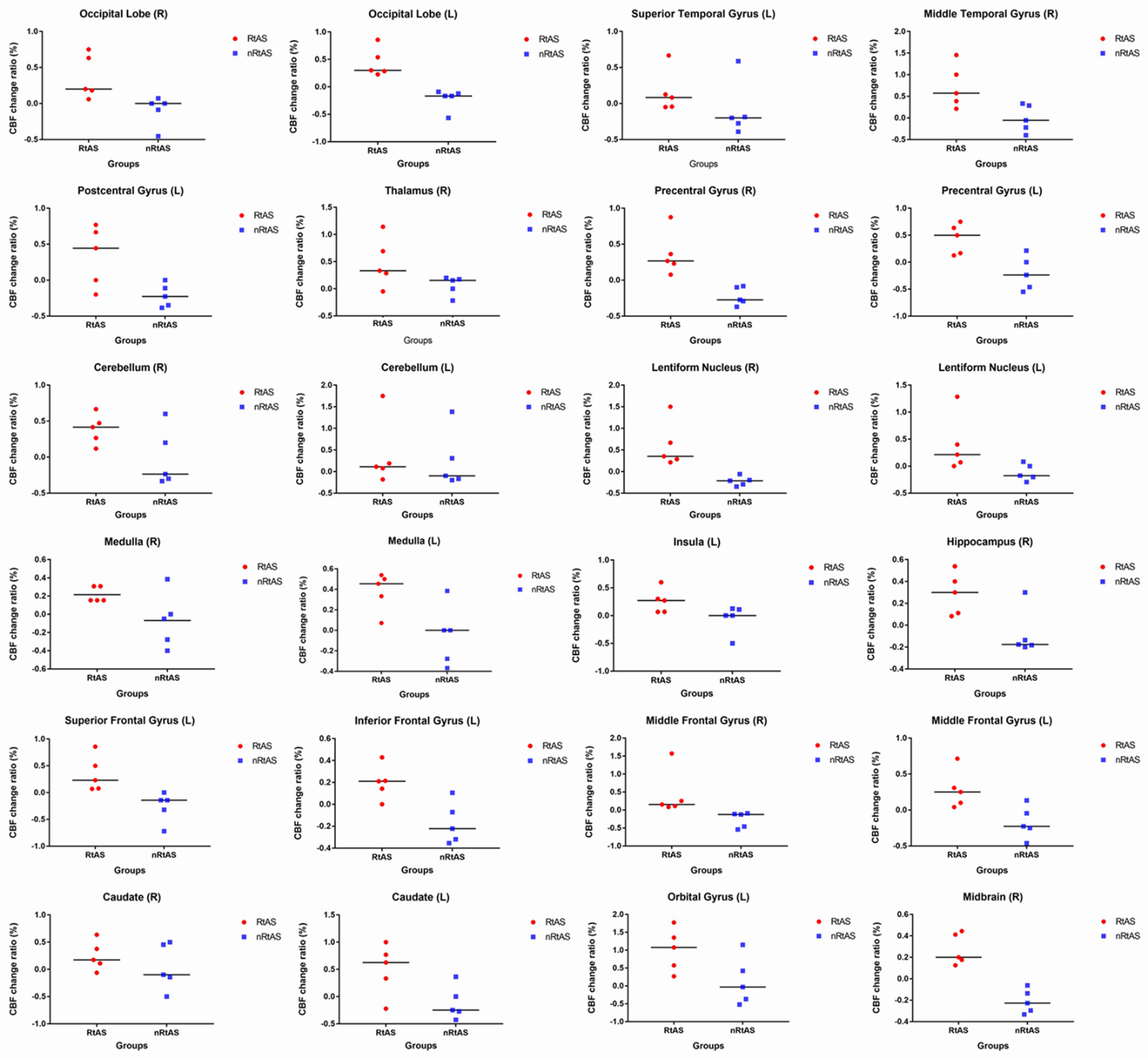
**

**Supplementary Figure 2 CBF changes ratio graphs**

**Supplementary Table 1**

**ROI-based CBF changes between pre-taVNS and post-taVNS in the RtAS group**

| ROIs | Pre-taVNS CBF (ml/100g/min) | Post-taVNS CBF (ml/100g/min) | F | P |
| --- | --- | --- | --- | --- |
| Thalamus | 12.54 | 16.46 | 14.61 | 0.06 |
| Medulla | 15.83 | 20.46 | 0.17 | 0.72 |
| Insula | 12.31 | 15.25 | 0.02 | 0.89 |
| Prefrontal areas | 12.96 | 16.84 | 0.17 | 0.72 |
| Middle temporal gyrus | 13.89 | 20.31 | 0.31 | 0.64 |
| Superior temporal gyrus | 13.37 | 16.89 | 0.82 | 0.46 |
| Caudate | 13.46 | 21.31 | 1.49 | 0.35 |
|  |  |  |  |  |
